# Microvascular resistance reserve: insights into discordant resting and hyperemic epicardial coronary physiology

**DOI:** 10.3389/fcvm.2026.1929694

**Published:** 2026-10-14

**Authors:** Aswin Babu, Isobelle Goldrick, Billy Broadbent, Rania Faeq, Charley Budgeon, Roopa Krishnamoorthy, Primero Ng, Cara Barnes, Dipen Sankhesara, Avadhesh Saraswat, Imran Shiekh, James Marangou, Richard Alcock, Rachel Orme, Carl Schultz, Graham S. Hillis, Jon Spiro

**Affiliations:** 1 Department of Cardiology, Royal Perth Hospital, Perth, WA, Australia; 2 School of Medicine, University of Western Australia, Perth, WA, Australia

**Keywords:** coronary flow reserve, coronary microvascular dysfunction, discordance, fractional flow reserve, microvascular resistance reserve, resting full-cycle ratio

## Abstract

**Introduction:**

Discordance between resting and hyperemic epicardial physiology is commonbut mechanistically unclear. Microvascular resistance reserve (MRR), a novelindex of vasodilatory capacity, may provide insight. We aimed to characterizethe prevalence and microvascular substrate of FFR/RFR discordance, andwhether MRR is independently associated with discordant phenotypes.

**Methods:**

We retrospectively analyzed 891 vessels from 723 consecutive patients with paired resting full-cycle ratio (RFR) and fractional flow reserve (FFR) measurements (June 2018–October 2024). Vessels were categorized as concordant or discordant. An operator-selected subset of 210 underwent coronary flow reserve (CFR), index of microvascular resistance (IMR), and MRR assessment. Multivariable logistic regression identified predictors of each discordant phenotype (FFR−/RFR+ and FFR+/RFR−).

**Results:**

Discordance was present in 195 vessels (22%); 121 (13.6%) FFR−/RFR+ and 74 (8.3%) FFR+/RFR−. Discordance was more common in the LAD (25% vs. 17%, OR 1.68, 95% CI 1.20–2.36, *p* = 0.003), driven predominantly by the FFR−/RFR+ phenotype (OR 2.43, 95% CI 1.56–3.78, *p* < 0.001). In the microvascular subset (*n* = 210), reduced CFR (OR 0.77, 95% CI 0.59–0.97, *p* = 0.039) and reduced MRR (OR 0.81, 95% CI 0.65–0.97, *p* = 0.037) were independently associated with the FFR−/RFR+ phenotype, while neither predicted the FFR+/RFR− phenotype. However, among 45 discordant vessels with microvascular data, MRR provided greater discrimination than CFR (AUC 0.712, 95% CI 0.539–0.884 vs. 0.608, 95% CI 0.434–0.783, *p* = 0.018).

**Discussion:**

These exploratory, hypothesis-generating findings suggest that impaired microvascular vasodilatory reserve, expressed predominantly as elevated resting flow, may contribute mechanistically to the FFR−/RFR+ phenotype.

## Introduction

Measurement of hyperemic and resting epicardial physiology is currently recommended to help guide management decisions among patients undergoing invasive coronary assessment (1–4). Although frequently used as separate and interchangeable assessments, hyperemic and resting epicardial indices may frequently disagree with discordance reported in up to one fifth of contemporary studies (5–8). The mechanism responsible for discordance and the clinical relevance of this observation remain poorly understood (6–8).

In the presence of epicardial coronary artery disease, autoregulation of coronary artery blood flow may occur in order to preserve adequate myocardial perfusion which may result in increased flow at rest. Additionally, disturbance in the health of the coronary microcirculation, manifest as impairment of vasodilatory capacity, may compromise the microcirculation's ability to respond and vasodilate to a hyperemic trigger (2, 6, 7). Recently, microvascular resistance reserve (MRR) has been described and validated as an accurate measurement of the vasodilatory capacity of the coronary microcirculation, independent of epicardial pressure loss (9). In brief, MRR is calculated as the ratio of CFR to FFR, corrected for forward driving (aortic) pressures during rest and hyperemia. Hence, by adjusting CFR for the pressure loss elicited by epicardial disease, MRR represents a microcirculatory specific index that describes the extent to which hyperemic microvascular resistance would fall if the epicardial artery were normal, such that a higher MRR indicates a greater vasodilatory capacity of the microcirculation.

MRR may therefore provide a unique and useful insight into the functional ability of the microcirculation and, when measured at the same time as epicardial assessment, mechanisms responsible for discordant resting and hyperemic epicardial physiology.

## Materials and methods

### Study design and patient population

This retrospective, single-center, observational study reports data from consecutive patients who underwent invasive coronary physiology assessment at our center between June 2018 and October 2024. Inclusion criteria: (i) paired FFR and RFR measurements available from the same coronary artery location and (ii) absence of significant transducer drift (defined as Pd/Pa ≥ 0.02 on pullback). Exclusion criteria: (i) concurrent enrollment in another clinical trial and (ii) studies performed for elective evaluation of ischemia with non-obstructive coronary arteries (INOCA) (Supplementary Figure S1). All coronary physiology data were reviewed by two cardiologists (JS, AB) experienced in performing and interpreting invasive coronary physiology studies. Ambiguous findings or discrepancies were resolved by consensus, involving a third cardiologist as required.

### Study aims

In the present study, we aimed to (a) define the prevalence of FFR/RFR discordance, (b) identify clinical, angiographic and territorial associations and (c) determine whether microvascular physiology, specifically coronary flow reserve (CFR) and MRR, was independently associated with discordant phenotypes.

### Ethical consideration

The study was approved by local governance committee, providing institutional approval for retrospective analysis of routinely collected clinical data and was conducted in accordance with the principles of the Declaration of Helsinki.

### Invasive coronary physiology

All physiology studies were performed using a PressureWire X (Abbott Vascular, California, USA) via a 6 Fr guiding catheter, following administration of intracoronary nitrate and appropriate zeroing and equalization. RFR was recorded at rest over a minimum of four cardiac cycles, with FFR recorded during intravenous adenosine-induced hyperemia (initial infusion 140 µg/kg/min, with operator-directed escalation when required). Additionally, indices of microvascular function including coronary flow reserve (CFR) and index of microcirculatory resistance (IMR) were obtained by bolus thermodilution (BTh) using three 3 mL injections of room-temperature saline at rest and repeated during hyperemia. MRR was calculated offline using data acquired from above. Transducer drift was assessed at the end of each study, with studies demonstrating P_d_/P_a_ drift ≥0.02 being excluded. Definitions, derivations and interpretive thresholds for all coronary physiology indices are summarized in Supplementary Table S1.

### Epicardial indices

RFR was taken as the lowest P_d_/P_a_ averaged over a minimum of four cardiac cycles at rest, with RFR ≤0.89 considered positive. FFR was taken as the ratio of distal coronary to aortic pressure during maximal intravenous adenosine-induced hyperemia, with FFR ≤0.80 considered positive (1). Following acquisition of paired measurements, vessels were categorized into four groups: Group 1 concordant positive (FFR+/RFR+); Group 2 discordant FFR+ (FFR+/RFR−); Group 3 discordant RFR+ (FFR−/RFR+); Group 4 concordant negative (FFR−/RFR−).

### Microvascular indices

CFR (Mean T_mn,rest_/Mean T_mn,hyper_) and IMR (T_mn,hyper_ × P_d,hyper_) were derived from BTh as previously described (10, 11). CMD was defined as low CFR (<2.5) and/or elevated IMR (≥25), with three distinct endotypes: (i) structural CMD (CFR <2.5 with IMR ≥25); (ii) functional CMD (CFR <2.5 with IMR <25) and (iii) isolated IMR elevation (CFR ≥2.5 with IMR ≥25) (1).

### Microvascular resistance reserve

The derivation of MRR has been previously described in detail (9). Although originally described using continuous thermodilution (CTh), MRR may also be derived from BTh. As mentioned above, MRR may be calculated by: (CFR/FFR) × (P_a,rest_/P_a,hyper_) where division by FFR corrects for epicardial pressure loss, rendering the index theoretically independent of epicardial stenosis severity (9). In addition, we calculated the basal microvascular resistance (BMR) in two forms: (i) real BMR (P_d,rest_ × T_mn,rest_) and (ii) theoretical BMR (P_a,rest_ × T_mn,rest_), the latter representing the resting resistance if no epicardial disease was present. The resistive reserve ratio (RRR = real BMR/IMR) values were taken as recorded at the time of the physiology study (12).

### Study endpoints

The primary endpoint was prevalence of FFR/RFR discordant physiology, either FFR+/RFR− or FFR−/RFR+. Secondary endpoints included the diagnostic performance of RFR for detecting FFR ≤0.80 and the association of FFR/RFR discordance with (i) clinical presentation and patient comorbidities, (ii) lesion territory and location, and (iii) microvascular physiology (CMD prevalence, CMD endotype, CFR, IMR, BMR, RRR and MRR) when available.

### Statistical analyses

Continuous variables are presented as mean (SD) or median (IQR, Q1–Q3) as appropriate, and categorical data as frequency (%). Continuous variables were compared using Student's *t*-test for normally distributed data and Mann–Whitney *U* for skewed data, with normality assessed using the Shapiro–Wilk test. For comparisons across more than two groups, the Kruskal–Wallis test was used. Categorical variables were compared using Chi-squared tests (*χ*^2^) or Fisher's exact tests, as appropriate. Correlation between RFR and FFR was assessed using Pearson's correlation coefficient (*r*), and receiver operating characteristic (ROC) curve analysis was performed to evaluate the diagnostic performance of RFR to detect an abnormal FFR (≤0.80), with the optimal RFR threshold determined using the Youden criterion.

To identify predictors of discordance, univariable logistic regression was first performed for each patient and vessel-level variable. Per-phenotype multivariable logistic regression models (FFR−/RFR+ and FFR+/RFR− vs. concordant vessels) were pre-specified with the limited covariates of age, sex and hypertension, with specific microvascular indices (CFR and MRR) entered one at a time given the mathematical collinearity of the indices. An exploratory all-indices multivariable model that simultaneously enters CFR and MRR together with the full clinical covariate set is reported as a sensitivity analysis (Supplementary Table S8). Real and theoretical BMR, RRR and MRR were compared between discordant phenotypes and in an exploratory model restricted to discordant vessels, IMR and theoretical BMR were entered simultaneously into a logistic regression model for the FFR−/RFR+ phenotype, with both terms standardized so that odds ratios are expressed per standard deviation increase. Odds ratios (OR) are presented with 95% confidence intervals (CI), and a two-sided *P* value <0.05 was considered statistically significant. Receiver operating characteristic (ROC) curves were constructed to assess the discriminative ability of CFR and MRR for each discordant phenotype, with the area under the curve (AUC) reported alongside 95% confidence intervals. Pairwise comparisons between AUCs were performed using the DeLong test. All analyses were performed using R (version 4.4.1, R Foundation for Statistical Computing, Vienna, Austria) (13). A full description of the pre-specified vs. exploratory analysis plan is provided in the Supplementary Methods.

## Results

### Patient characteristics

During the study period, coronary physiology studies were performed in 1,328 vessels (1,038 patients), with 382 vessels excluded due to absence of paired FFR/RFR data, 66 excluded due to concurrent enrollment of the patient in another clinical trial and 57 vessels excluded due to planned INOCA testing (68 vessels met more than one exclusion criterion), with the remaining 891 vessels (723 patients) representing our study cohort (Supplementary Figure S1).

Patient characteristics are summarized in Table 1; median age 65 years (IQR, 58–73), 76% male. In addition, 366 patients (51%) were investigated for stable coronary artery disease, 208 (29%) acute coronary syndromes (ACS) and the remaining 20% underwent coronary angiography for other indications such as work-up for valvular heart disease and heart failure of unknown etiology. Patients demonstrated a high prevalence of cardiovascular risk factors with a similar distribution between groups (Table 1).

**Table 1:** Patient characteristics stratified by discordance (paired RFR/FFR cohort).

Variable	Overall (*N* = 723)	Concordant only (*N* = 538)	≥1 discordant vessel (*N* = 185)	*p*-value
Age, years	65 [58–73]	65 [58–72]	66 [59–73]	0.501
Sex				0.726
Male	552 (76%)	413 (77%)	139 (75%)	
Female	171 (24%)	125 (23%)	46 (25%)	
Presentation				
ACS	208 (29%)	156 (29%)	52 (28%)	0.892
Staged STEMI	42 (20%)	34 (22%)	8 (15%)	
NSTEMI	114 (55%)	82 (53%)	32 (62%)	
Unstable angina	32 (15%)	24 (15%)	8 (15%)	
Unspecified troponin-positive chest painᵃ	20 (10%)	16 (10%)	4 (8%)	
Elective: chest pain investigation	366 (51%)	273 (51%)	93 (50%)	0.979
Hypertension	477 (66%)	344 (64%)	133 (72%)	0.060
Diabetes mellitus	246 (34%)	182 (34%)	64 (35%)	0.921
Smoker (current/ex)	378 (52%)	275 (51%)	103 (56%)	0.324
Dyslipidemia	381 (53%)	291 (54%)	90 (49%)	0.233
eGFR, mL/min/1.73 m^2^	85 [70–90]	85 [70–90]	87 [71–90]	0.386
LVEF, %	55 [50–62]	55 [50–62]	55.5 [50.5–62]	0.557
Previous PCI	170 (24%)	127 (24%)	43 (23%)	1.000
Previous CABG	12 (1.7%)	6 (1.1%)	6 (3.2%)	0.087

Values are median [Q1–Q3] or *n* (%). Comparisons by Mann–Whitney *U*-test (continuous) or chi-squared or Fisher exact test (categorical), as appropriate.

ᵃPatients with a dynamic troponin rise in the context of ventricular arrhythmia, decompensated heart failure, or severe valvular heart disease.

ACS, acute coronary syndrome; CABG, coronary artery bypass grafting; eGFR, estimated glomerular filtration rate; LVEF, left ventricular ejection fraction; NSTEMI, non-ST-elevation myocardial infarction; PCI, percutaneous coronary intervention; STEMI, ST-elevation myocardial infarction.

### FFR/RFR discordance

Within our cohort, FFR/RFR discordance was present in 22% (*n* = 195) of studies; positive RFR discordance (FFR−/RFR+) in 121 (13.6%) and positive FFR discordance (FFR+/RFR−) in 74 (8.3%) of studies (Supplementary Table S2). Concordant normal (FFR−/RFR−) or abnormal (FFR+/RFR+) results were present in the remaining 696 studies (78.1%). FFR and RFR demonstrated a moderate-to-strong correlation (*r* = 0.717, *p* < 0.001), with RFR ≤0.89 demonstrating an AUC of 0.861 (95% CI 0.837–0.886) in predicting a positive FFR (≤0.80) (Figure 1 and Supplementary Figure S2). The Youden optimal threshold was an RFR of 0.895, demonstrating identical sensitivity and specificity to the 0.89 cut off (Sensitivity: 78% and Specificity: 78% respectively).

**Figure 1:**
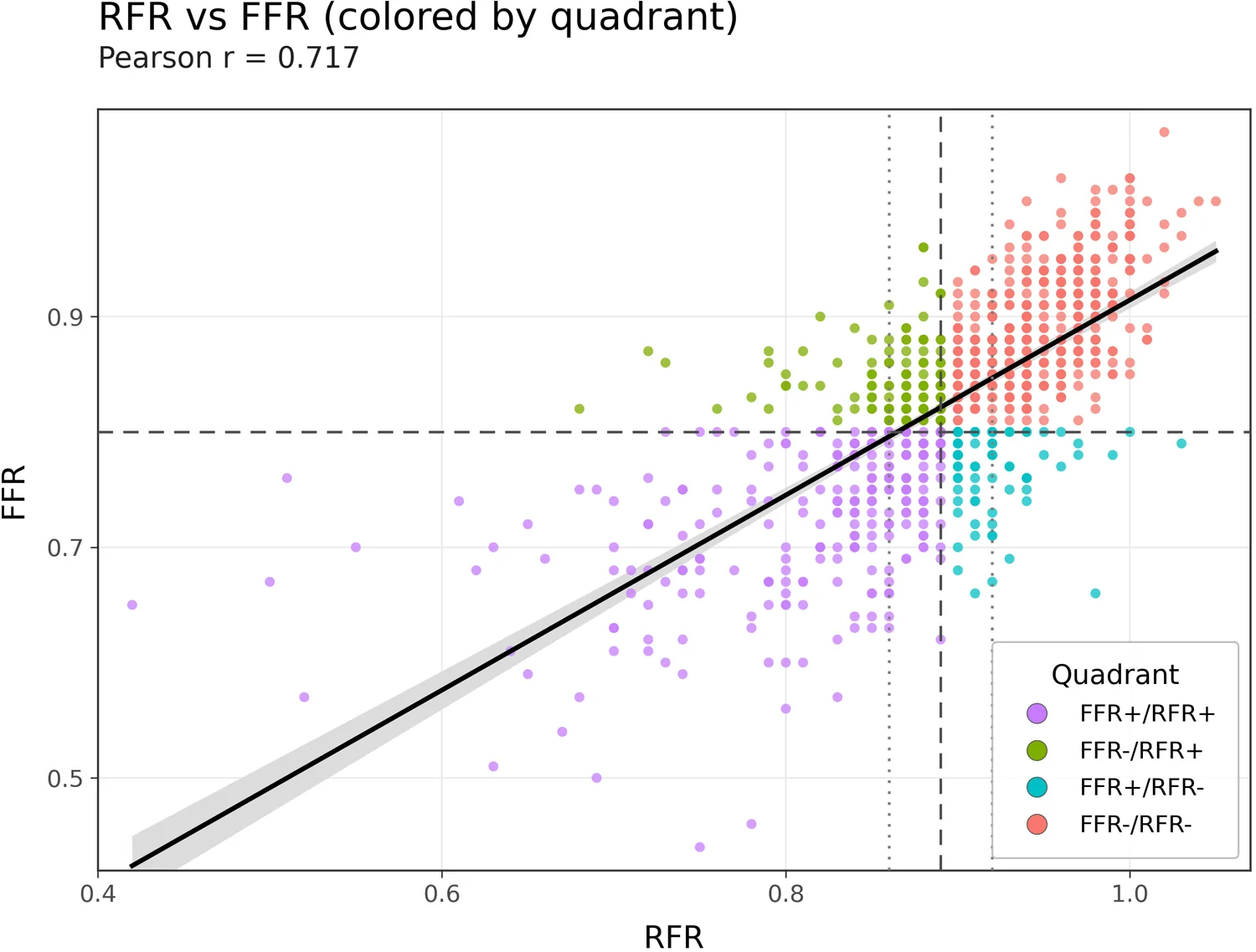
Scatter plot of RFR versus FFR colored by concordance quadrant. Each point represents one vessel (*N* = 891). Dashed lines indicate the decision thresholds (FFR ≤0.80, RFR ≤0.89). Quadrants correspond to concordant-abnormal (FFR+/RFR+), concordant-normal (FFR−/RFR−), FFR−/RFR+ (abnormal resting pressure ratio, normal hyperemic pressure ratio) and FFR+/RFR− (normal resting pressure ratio, abnormal hyperemic pressure ratio) Pearson correlation *r* = 0.717. FFR, fractional flow reserve; RFR, resting full-cycle ratio.

### Clinical and angiographic correlates of FFR/RFR discordance

Physiology studies were most commonly performed in the LAD (*n* = 528, 59%), with discordant findings being more frequently found in this vessel, compared with non-LAD studies (25% vs. 17%; OR 1.68, 95% CI 1.20–2.36; *p* = 0.003) (Supplementary Tables S3, S4). In particular, testing within the LAD territory was associated with a significantly higher prevalence of the RFR positive discordant phenotype (FFR−/RFR+) compared with non-LAD vessels (OR 2.43, 95% CI 1.56–3.78, *p* < 0.001) but not the FFR positive discordance phenotype (FFR+/RFR−), OR 0.89, 95% CI 0.55–1.45, *p* = 0.647 (Supplementary Table S4 and Figure 2). Among ACS patients undergoing invasive coronary physiology, where data for the culprit/non-culprit vessel was clearly available (*n* = 97/248), 70% of physiology studies were performed within the non-culprit vessel. Overall, rates of discordant findings were similar between patients with ACS and non-ACS presentations (21.4% vs. 22.1%, *p* = 0.818).

**Figure 2:**
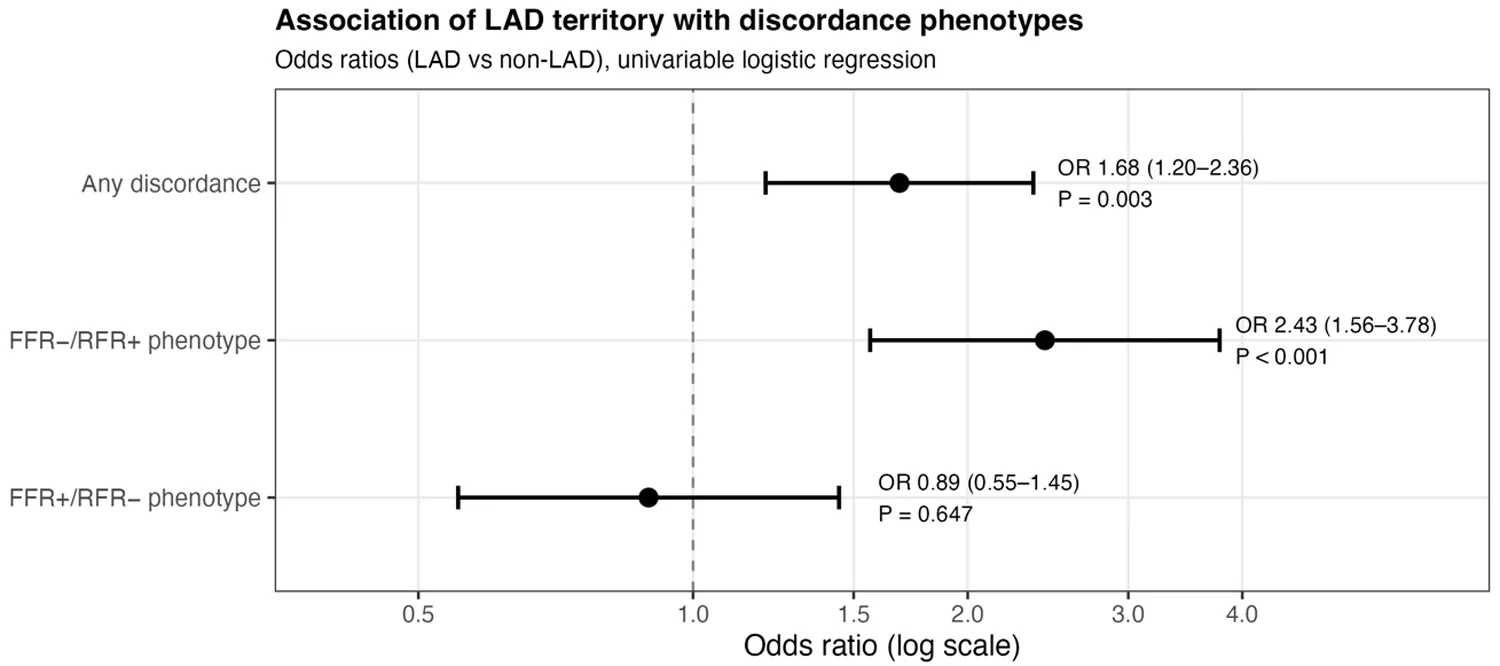
Forest plot of the association between LAD territory and discordance phenotypes. Odds ratios (LAD versus non-LAD) from univariable logistic regression for any discordance, the FFR−/RFR+ phenotype, and the FFR+/RFR− phenotype. Any discordance and the FFR−/RFR+ phenotype were both significantly associated with the LAD (OR 1.68, 95% CI 1.20–2.36, *p* = 0.003, and OR 2.43, 95% CI 1.56–3.78, *p* < 0.001, respectively), whereas the FFR+/RFR− phenotype was not (OR 0.89, 95% CI 0.55–1.45, *p* = 0.647). CI, confidence interval; FFR, fractional flow reserve; LAD, left anterior descending; OR, odds ratio; RFR, resting full-cycle ratio.

### Impact of microvascular dysfunction

Among our study cohort, CMD testing was performed at the same time as FFR/RFR measurement in 210 vessels (24% of all physiology studies) in 183 patients; median CFR 3.34 (IQR, 2.23–4.98), IMR 13.66 (IQR, 9.25–20.89) and MRR 4.25 (IQR, 2.89–6.01) (Table 2). Within vessels undergoing CMD testing, FFR/RFR discordance was present in 21.4% (45 studies). Among patients with available CMD data, the presence of CMD was similar among patients with concordant and discordant epicardial physiology (38.8% vs. 42.2% respectively, OR 1.15, 95% CI 0.55–2.37, *p* = 0.732) (Supplementary Table S5). However, among the FFR−/RFR+ phenotype specifically, CMD prevalence was numerically higher (47%) than the concordant cohort (38.8%). Furthermore, there was a trend towards structural CMD in the FFR−/RFR+ phenotype when compared to the concordant group (18.8% vs. 7.3%, OR 2.92, 95% CI 0.82–9.35, *p* = 0.085) (Supplementary Table S5 and Figure S3). Across all quadrants of possible FFR/RFR results, CFR differed significantly (*p* = 0.049) with lower values observed in quadrants with abnormal RFR (FFR−/RFR+ and FFR+/RFR+) compared to those with normal RFR (Supplementary Table S6), whereas IMR and MRR did not differ significantly (*p* = 0.572 and *p* = 0.155 respectively). Compared with the FFR+/RFR− discordant phenotype, both CFR and MRR were lower among patients with FFR−/RFR+ discordance (CFR: 2.89 [IQR, 1.80–4.02] vs. 3.75 [IQR, 2.80–3.97]; MRR: 3.89 [IQR, 2.42–4.83] vs. 4.99 [IQR, 4.21–7.13]) (Figure 3). MRR also differentiated the FFR−/RFR+ phenotype in unadjusted comparison (*p* = 0.028), and ROC analysis demonstrated that MRR provided greater discrimination for the FFR−/RFR+ phenotype than CFR (AUC 0.712, 95% CI 0.539–0.884 vs. 0.608, 95% CI 0.434–0.783, *p* = 0.018) (Figure 4). The independent association of both CFR and MRR with the FFR−/RFR+ phenotype is further explored in the regression analyses below (Table 3 and Supplementary Table S7).

**Table 2:** Baseline angiographic and procedural characteristics (vessel-level cohort, *N* = 891).

Characteristic	Overall (*N* = 891)	Concordant (*N* = 696)	FFR−/RFR+ (*N* = 121)	FFR+/RFR− (*N* = 74)	*p*-value
Angiographic characteristics					
Artery tested					<0.001
LAD	528 (59%)	394 (57%)	92 (76%)	42 (57%)	
LCx	110 (12%)	94 (14%)	9 (7.4%)	7 (9.5%)	
RCA	159 (18%)	131 (19%)	8 (6.6%)	20 (27%)	
LM	42 (4.7%)	33 (4.7%)	6 (5.0%)	3 (4.1%)	
Other	51 (5.7%)	44 (6.3%)	6 (5.0%)	1 (1.4%)	
Graft	1 (0.1%)	0 (0%)	0 (0%)	1 (1.4%)	
Lesion location (*n* = 233 with data)					0.490
Proximal	79 (34%)	61 (35%)	10 (42%)	8 (22%)	
Mid	134 (58%)	97 (56%)	13 (54%)	24 (67%)	
Distal	20 (8.6%)	15 (8.7%)	1 (4.2%)	4 (11%)	
Procedural context (ACS vessels with target data, *n* = 97)					0.072
Culprit lesion	29 (30%)	18 (28%)	2 (14%)	9 (50%)	
Non-culprit lesion	68 (70%)	47 (72%)	12 (86%)	9 (50%)	
Epicardial physiology (*n* = 891 vessels)					
FFR	0.84 [0.78–0.89]	0.85 [0.77–0.90]	0.84 [0.82–0.86]	0.78 [0.75–0.79]	<0.001
RFR	0.91 [0.86–0.95]	0.92 [0.86–0.96]	0.87 [0.85–0.89]	0.92 [0.90–0.93]	<0.001
Microvascular physiology (*n* = 210 vessels)					
CFR	3.34 [2.23–4.98]	3.39 [2.25–5.16]	2.89 [1.80–4.02]	3.75 [2.80–3.97]	0.123
IMR	13.66 [9.25–20.89]	13.25 [9.15–20.55]	13.72 [9.56–21.57]	16.29 [12.00–21.00]	0.871
MRR	4.25 [2.89–6.01]	4.22 [3.00–6.47]	3.89 [2.42–4.83]	4.99 [4.21–7.13]	0.073
RRR	4.15 [2.89–5.90]	4.22 [2.95–6.22]	3.74 [2.26–4.54]	4.79 [4.30–7.30]	0.048
Real BMR, mmHg·s	60.19 [35.40–89.37]	62.61 [35.82–93.13]	42.57 [32.90–70.26]	70.57 [50.63–94.89]	0.064
Theoretical BMR, mmHg·s	63.20 [38.10–93.91]	65.15 [38.43–97.10]	46.52 [36.24–75.65]	73.83 [54.75–95.69]	0.088

Values are median [Q1–Q3] or *n* (%). Comparisons by Kruskal–Wallis test (continuous) or chi-squared or Fisher exact test (categorical), as appropriate. A single overall chi-squared/Fisher exact test is reported for each grouped categorical variable.

ACS, acute coronary syndrome; BMR, basal microvascular resistance; CFR, coronary flow reserve; FFR, fractional flow reserve; IMR, index of microcirculatory resistance; LAD, left anterior descending; LCx, left circumflex; LM, left main; MRR, microvascular resistance reserve; RCA, right coronary artery; RFR, resting full-cycle ratio; RRR, resistive reserve ratio; Tmn, mean transit time.

**Figure 3:**
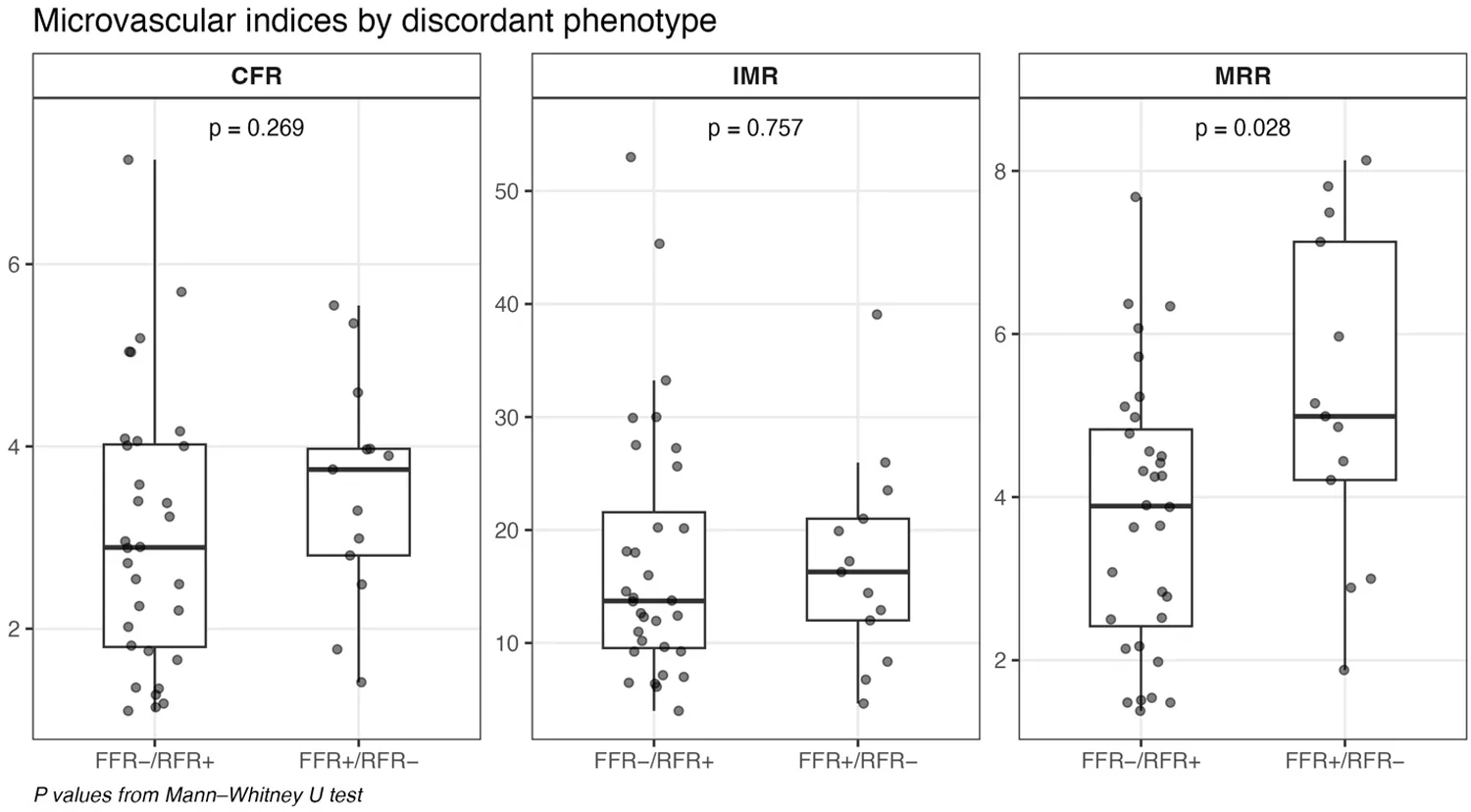
Box plots of microvascular indices (CFR, IMR, and MRR) in the two discordant phenotypes. Comparisons are shown for the 45 vessels with discordant epicardial physiology that also had paired microvascular data. MRR was significantly lower in the FFR−/RFR+ group compared with the FFR+/RFR− group (median 3.89 [IQR 2.42–4.83] vs 4.99 [IQR 4.21–7.13], *p* = 0.028), whereas CFR (*p* = 0.269) and IMR (*p* = 0.757) did not differ significantly between the two discordant phenotypes. *P* values from Mann–Whitney *U*-test. CFR, coronary flow reserve; FFR, fractional flow reserve; IMR, index of microcirculatory resistance; IQR, interquartile range; MRR, microvascular resistance reserve; RFR, resting full-cycle ratio.

**Figure 4:**
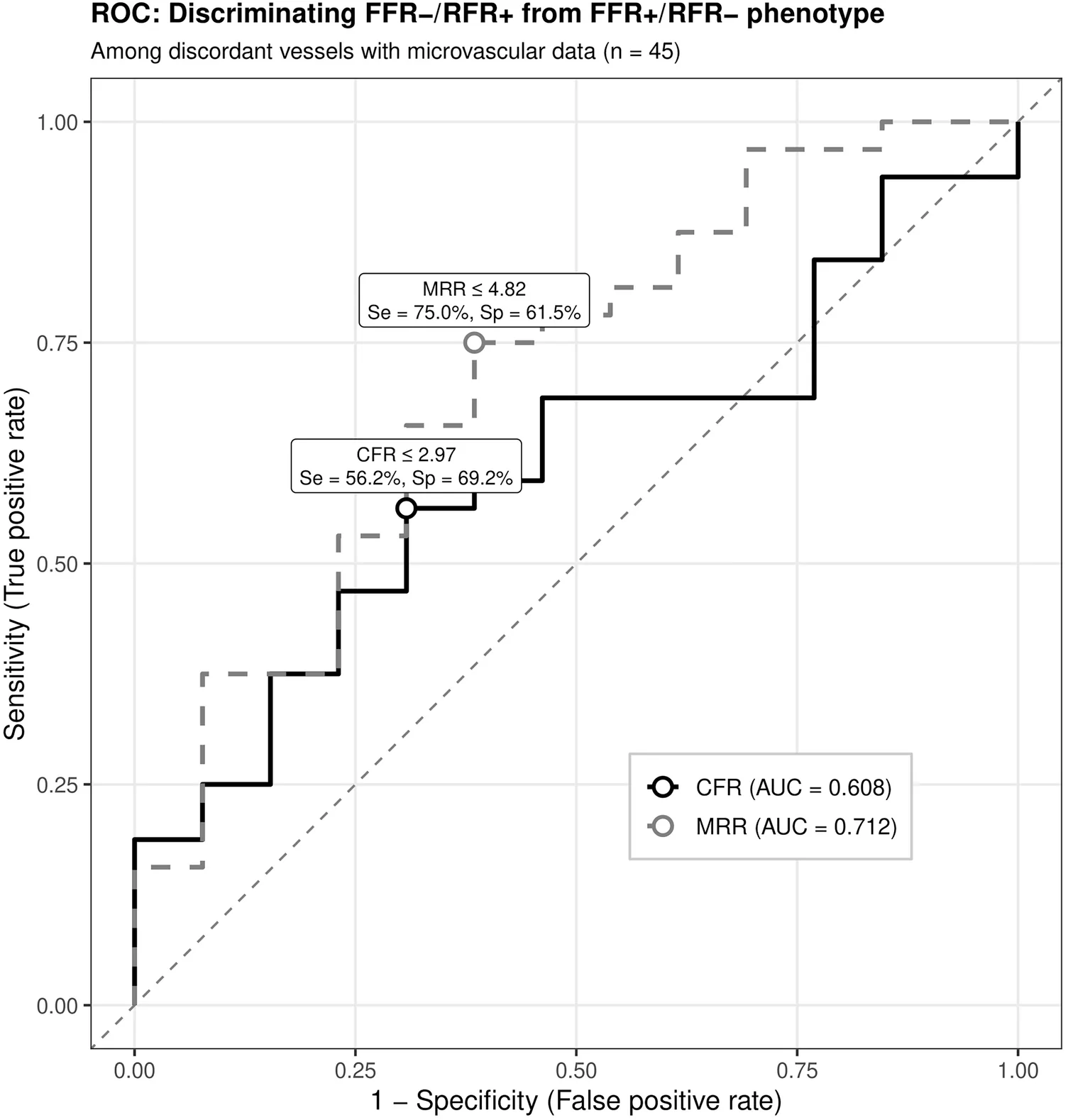
Receiver operating characteristic curves comparing MRR and CFR for discriminating the FFR−/RFR+ from the FFR+/RFR− phenotype among discordant vessels with microvascular data (*n* = 45). MRR (AUC 0.712, 95% CI 0.539–0.884, optimal threshold ≤4.82, sensitivity 75.0%, specificity 61.5%) outperformed CFR (AUC 0.608, 95% CI 0.434–0.783, optimal threshold ≤2.97, sensitivity 56.2%, specificity 69.2%) for phenotype discrimination, *p* = 0.018. AUC, area under the curve; CFR, coronary flow reserve; CI, confidence interval; FFR, fractional flow reserve; MRR, microvascular resistance reserve; RFR, resting full-cycle ratio.

**Table 3:** Per-phenotype multivariable logistic regression: predictors of discordant phenotypes (prespecified parsimonious models).

Model	Predictor	OR	95% CI	*P* value
FFR−/RFR+ vs concordant (CFR model)	Age (per year)	1.00	0.96–1.05	0.823
	Male sex	0.95	0.42–2.28	0.911
	Hypertension	1.10	0.49–2.59	0.821
	CFR (per 1-unit)	0.77	0.59–0.97	**0.039**
FFR−/RFR+ vs concordant (MRR model)	Age (per year)	1.00	0.96–1.05	0.936
	Male sex	0.94	0.42–2.24	0.885
	Hypertension	1.10	0.49–2.60	0.822
	MRR (per 1-unit)	0.81	0.65–0.97	**0.037**
FFR+/RFR− vs concordant (CFR model)	Age (per year)	1.01	0.94–1.08	0.813
	Male sex	5.48	1.00–102.48	0.111
	Hypertension	2.02	0.56–9.72	0.317
	CFR (per 1-unit)	0.87	0.61–1.17	0.407
FFR+/RFR− vs concordant (MRR model)	Age (per year)	1.02	0.95–1.09	0.597
	Male sex	5.00	0.91–93.54	0.132
	Hypertension	1.85	0.52–8.68	0.375
	MRR (per 1-unit)	1.04	0.82–1.27	0.727

CFR, coronary flow reserve; CI, confidence interval; FFR, fractional flow reserve; IMR, index of microcirculatory resistance; MRR, microvascular resistance reserve; OR, odds ratio; RFR, resting full-cycle ratio.

Each microvascular index (CFR & MRR) was entered in a separate pre-specified model together with age, sex, and hypertension, given the mathematical collinearity of CFR and MRR. The wide confidence intervals for the male sex coefficient in the FFR+/RFR− models reflect the small number of events in this phenotype (*n* = 13) and are indicative of parameter instability rather than a reliable effect estimate and should be interpreted accordingly.

In all models, the reference category is the combined concordant group (FFR+/RFR+ together with FFR−/RFR−).

Bold values indicate statistically significant associations (*p* < 0.05).

At the optimal Youden threshold, MRR ≤4.82 yielded a sensitivity of 75.0% and specificity of 61.5%, compared with CFR ≤2.97 (sensitivity 56.2%, specificity 69.2%) (Figure 4).

### Regression analysis

#### All discordant studies

Using univariable logistic regression, reduced CFR was associated with the presence of a discordant phenotype (OR 0.81 per 1-unit increase in CFR, 95% CI 0.66–0.99, *p* = 0.038), whereas MRR and IMR were not (Supplementary Table S7). In an exploratory sensitivity analysis entering CFR, MRR and IMR simultaneously in a multivariable regression model, CFR was associated with any discordance (OR 0.51 per 1-unit increase, 95% CI 0.28–0.90, *p* = 0.021) (Supplementary Table S8).

#### FFR−/RFR+ discordant studies

Univariable modeling demonstrated that increased CFR and MRR corresponded with a reduction in the FFR−/RFR+ phenotype; CFR 23% reduction per unit increase (OR 0.77, 95% CI 0.60–0.98, p = 0.034), MRR 20% reduction per unit increase (OR 0.80, 95% CI 0.66–0.97, p *=* 0.025) (Supplementary Table S7). Older age (OR 1.04 per year, 95% CI 1.02–1.06, *p* < 0.001) and hypertension (OR 1.68, 95% CI 1.08–2.64, *p* = 0.023) were significantly associated with the FFR−/RFR+ phenotype while being male was associated with a lower prevalence (OR 0.60, 95% CI 0.40–0.92, *p* = 0.018). Due to the low rates of discordance in the subset with available microvascular physiology, and the mathematical overlap between CFR and MRR, two separate multivariable models were used with minimal clinical covariates (age, sex, hypertension) (Table 3). Multivariable analysis suggested that both lower CFR and MRR were independently associated with the presence of FFR−/RFR+ discordance; OR 0.77, 95% CI 0.59–0.97, *p* = 0.039 and OR 0.81, 95% CI 0.65–0.97, *p* = 0.037 respectively (Table 3). Intriguingly, no such association for the FFR+/RFR− discordant phenotype was demonstrated using the same analyses (Supplementary Table S7 and Table 3).

#### Basal microvascular resistance and resistive reserve ratio

Both theoretical and real BMR were calculated in all 210 vessels that underwent microvascular assessment. Among discordant vessels, real and theoretical BMR were numerically lower in the FFR−/RFR+ group compared to the FFR+/RFR− group (real BMR: 42.57 [IQR, 32.90–70.26] vs. 70.57 [IQR, 50.63–94.89] mmHg·s, *p* = 0.059; theoretical BMR: 46.52 [IQR, 36.24–75.65] vs. 73.83 [IQR, 54.75–95.69] mmHg·s, *p* = 0.062) and was associated with a numerical increase in resting flow (FFR−/RFR+ mean T_mn,rest_: 0.60 [IQR, 0.41–0.80] vs. FFR+/RFR− 0.80 [IQR, 0.63–0.97] seconds, *p* = 0.101) (Supplementary Table S6). Mean hyperemic transit times were similar with no difference in IMR between the groups (Supplementary Table S6). In agreement with MRR (*p* = 0.028), RRR also separated the discordant phenotypes (3.74 [IQR, 2.26–4.54] vs. 4.79 [IQR, 4.30–7.30], *p* = 0.018) (Supplementary Table S6). In addition, an exploratory model combining theoretical BMR and IMR demonstrated that only theoretical BMR was associated with the FFR−/RFR+ phenotype (OR 0.41 per SD, 95% CI 0.17–0.98, *p* = 0.045; IMR OR 1.70 per SD, 95% CI 0.66–4.36, *p* = 0.270) (Supplementary Table S9).

## Discussion

In this large cohort of consecutive patients undergoing invasive coronary physiology, discordance of epicardial physiology (FFR/RFR) occurred in approximately one in five vessels, in keeping with previous reports (6–8). Discordant epicardial physiology was predominantly represented by the FFR−/RFR+ phenotype (62.1% of all discordance) and was most commonly observed among LAD studies.

In our study, while CFR predicted the presence of discordant epicardial physiology, MRR demonstrated superior detection of the FFR−/RFR+ discordant phenotype compared to CFR. These observations may suggest that an impaired vasodilatory capacity of the microcirculation, as identified by low MRR, may be mechanistically important in explaining discordance of epicardial measurements.

### Autoregulation of coronary artery blood flow

In the presence of epicardial coronary artery disease, autoregulation may increase resting coronary blood flow, while the presence of impaired microvascular vasodilatory capacity may diminish hyperemic flow, leading to potential disagreement between resting and hyperemic measurements (14–16). Consistent with this, the FFR−/RFR+ phenotype was the most prevalent direction of discordance in our cohort and was associated with reduced CFR and low MRR. In particular, submaximal vasodilation at rest with elevated resting flow generating a resting trans-stenotic gradient may render the resting index positive while hyperemic FFR remains preserved.

Indeed, our comprehensive break down of resting (theoretical BMR) and hyperemic (IMR) components of MRR revealed that a lower theoretical BMR, and not an elevated IMR, was associated with the FFR−/RFR+ phenotype (OR 0.41 per SD vs. OR 1.70 per SD, *p* = 0.045). Furthermore, compared with FFR+/RFR− the FFR−/RFR+ phenotype demonstrated a trend towards shorter resting transit time despite similar hyperemic resistance (IMR). Therefore, both diminished CFR and MRR likely reflect autoregulatory microvascular dilation at rest, rather than an increase in hyperemic microvascular resistance, in keeping with computational data which suggests that a low BMR may drive a positive non-hyperemic pressure ratio (NHPR) (17). Consistent with this, Echavarría-Pinto et al. demonstrated that in patients with stable coronary artery disease with intermediate stenosis, FFR−/NHPR+ vessels (*n* = 35) demonstrated lower BMR when compared to concordantly normal vessels (35.9 vs. 55.2 mmHg·s) (18).

In keeping with this hypothesis, MRR, which represents a more precise index of microcirculatory vasodilatory reserve (9, 19), was significantly reduced in vessels with the FFR−/RFR+ phenotype, consistent with prior Doppler-derived data demonstrating reduced hyperemic flow velocity in the FFR−/NHPR+group (2, 16).

Importantly, reduced CFR may not be a benign finding even in the presence of reassuring FFR, with prior studies reporting hazard ratios between 3 and 5 for adverse cardiovascular outcomes (cardiac death and myocardial infarction) among patients with abnormal CFR and normal FFR (20–22). Accordingly, patients with reduced CFR and MRR within the FFR−/RFR+ phenotype may warrant closer clinical surveillance despite a reassuring FFR.

#### Microvascular resistance reserve (MRR)

Although CFR varied significantly across all four quadrants of FFR/RFR measurements, MRR significantly separated the discordant phenotypes. Indeed, median MRR was significantly lower in the FFR−/RFR+ phenotype compared with FFR+/RFR− group (3.89 vs. 4.99, *p* = 0.028). Additionally, while overall CMD prevalence and endotype distribution were not statistically different between the discordant groups, there was a numerical trend towards lower MRR in the FFR−/RFR+ cohort (MRR <3.0: 37.5% vs. 15.4%). Additionally, dichotomously defined CMD (CFR <2.5 and/or IMR ≥25) was also more prevalent in the FFR−/RFR+ cohort compared to the concordant vessels (47% vs. 38.8%). This direction closely mirrors Legutko et al., in whom CMD was present in 64% of discordant vessels compared to 41% of concordant vessels (6). Yet, both in this cohort and our study a substantial proportion of discordant vessels did not exhibit CMD, suggesting that discordance remains multifactorial with interplay between autoregulation, microvascular vasodilatory capacity and the presence of epicardial disease, not easily identified from binary CMD definitions alone.

The predictive ability of MRR may be further highlighted by regression modeling which identified MRR as an independent predictor for FFR-/RFR+ discordance with a 20% reduction in discordance for every unit increase in MRR. Therefore, while CFR may determine whether discordance occurs, MRR may help identify the direction of discordance.

Conceptually, this distinction is important as CFR represents a ratio determined by both resting and hyperemic flow and remains sensitive to pressure loss from epicardial disease. In contrast, MRR remains independent of epicardial pressure loss and is not influenced by changes in driving pressure or myocardial mass supplied therefore making it a potentially useful physiological index across all coronary territories (9, 23). Consequently, MRR represents a specific marker for the vasodilatory capacity of the microcirculation, with the ability to detect impaired vasodilatory reserve, whether arising from elevated resting flow or from a blunted hyperemic response. Consistent with this, in the present study among vessels with discordant epicardial measurements and CMD testing, MRR provided modest discrimination for identifying FFR−/RFR+ vessels (AUC 0.712, 95% CI 0.539–0.884, optimal threshold ≤4.82) and outperformed CFR (AUC 0.608, 95% CI 0.434–0.783, optimal threshold ≤2.97, *p* = 0.018). Although the FFR−/RFR+ phenotype was the most frequent discordant phenotype (62.1% of discordant studies), the AUC is a prevalence-invariant measure of discrimination, and the observed MRR AUC of 0.712 (95% CI 0.539–0.884) reflects discrimination above chance rather than a reflection of the skewed phenotype distribution alone. However, the proximity of the lower confidence bound to 0.5 reinforces the hypothesis-generating nature of this observation and the need for further validation.

Accordingly, our data suggest that MRR may help with phenotype characterization and detection of impaired microvascular vasodilatory reserve. Moreover, the utility of MRR in determining discordant epicardial physiology is not well established and further studies are required to better understand its clinical utility.

### Myocardial mass and perfusion territory

The LAD territory was associated with significantly higher discordance rates compared with non-LAD vessels (25.0% vs. 17.0%, OR 1.68, 95% CI 1.20–2.36, *p* = 0.003), driven predominantly by the FFR−/RFR+ phenotype (OR 2.43, 95% CI 1.56–3.78, *p* < 0.001), consistent with contemporary data identifying the LAD as an independent predictor of discordance (8, 24). This likely reflects the larger myocardial territory and greater vessel length of the LAD, which may amplify cumulative pressure loss from diffuse atherosclerosis and magnify coexisting microvascular dysfunction (20, 25, 26). Warisawa et al. further demonstrated the contribution of diffuse atherosclerosis in explaining FFR−/NHPR+ discordance with 82% of FFR−/NHPR+ vessels exhibiting a diffuse pattern of pressure loss in pressure-wire pullback analysis (27).

### Clinical implications

When resting and hyperemic indices disagree, outcome data favor an FFR-guided revascularization strategy, with discordance associated with higher adverse event rates compared with concordantly normal physiology (28, 29). Emerging data suggest that the revascularization benefit may be concentrated within the FFR+/NHPR− phenotype (focal flow-limiting disease with intact resistance reserve), whereas FFR−/NHPR+ lesions may reflect diffuse disease with impaired vasodilatory reserve where PCI may be less beneficial (27, 29). Although our findings are hypothesis generating, they suggest that adjunctive CMD testing while the pressure wire remains *in situ* may help identify abnormal microvascular physiology, guide treatment decisions and provide additional prognostic information at no additional cost. Furthermore, the novel pullback pressure gradient (PPG) analysis may further refine this distinction by characterizing the pattern of pressure loss as focal or diffuse, helping to identify patients in whom revascularization is most likely to be of benefit (30).

## Limitations

Several limitations should be acknowledged. Firstly, the retrospective single-center design is subject to selection bias and unmeasured confounding. In particular, the decision to perform paired FFR/RFR measurements and microvascular physiology (CMD testing) was at the discretion of the operator and may have preferentially selected patients with more diagnostically ambiguous or complex lesions. Secondly, microvascular physiology was only available in a subset of patients (*n* = 210 vessels) which resulted in a small number of studies with discordance that could report both epicardial and microvascular physiology (*n* = 45 vessels). This small sample size limited statistical power for phenotype comparisons and restricted the number of covariates that could be reliably used in multivariable logistic regression modeling for predicting discordance phenotype. Our findings should therefore be considered exploratory and hypothesis generating, highlighted by the wide confidence intervals reported for the discrimination of MRR. Thirdly, MRR in our study was derived using BTh rather than CTh. MRR derived using BTh may therefore be influenced by hemodynamic and technical variability. CTh provides an operator independent, absolute volumetric measurement of flow (mL/min) and resistance (Wood units, WU) with superior reliability (31–33) and has been validated against [^15^O]H_2_O positron emission tomography (PET) (34). Although BTh and CTh derived MRR correlate, the correlation is weak and BTh systematically produces higher values with greater variability (31) and the absence of a reference-standard comparator such as CTh or PET is a further limitation. Fourthly, detailed lesion-level phenotyping (length, percentage stenosis, focal vs. diffuse pattern, tandem lesions, calcium) was not captured, despite recognized associations with discordance, and could not be reliably reconstructed retrospectively. Moreover, inclusion of patients with ACS (29% of the cohort) may have introduced potential confounding, as both resting and hyperemic indices may be influenced by dynamic disturbance of microvascular function in the acute setting. Although discordance rates were similar between ACS and non-ACS presentations, the timing of physiology testing relative to the ACS event may have varied. The inclusion of ACS presentations reflects the all-comer design of the study, with 70% of ACS physiology studies performed in non-culprit vessels, in which pressure derived metrics remain more reliable as compared to measurements within the culprit vessel (12, 35). Additionally, impairment of left ventricular function may influence the findings of physiology studies. However, left ventricular ejection fraction (LVEF) was not documented in 26% of studies and within the microvascular subset (*n* = 210) only 16 vessels were from patients with a documented LVEF <50%, limiting meaningful interpretation. Beyond the microcirculation, it is also important to understand the contribution of autoregulation (highlighted by the difference in theoretical and real BMR), epicardial plaque characteristics and diffuse disease in FFR/RFR discordance. Finally, the absence of outcome data remains a limitation. However, given prior reports of elevated cardiovascular risk among patients with discordant epicardial physiology, our findings expand on the current literature and hypothesize that the microvasculature may represent a potential future therapeutic target.

## Future directions

As comprehensive coronary physiology testing, including assessment of the coronary microcirculation, becomes more commonplace several areas of uncertainty remain.

Prospective longitudinal studies are required to better understand the clinical outcome and management of patients with discordant epicardial physiology. Future work should incorporate lesion-level phenotyping, particularly in the LAD where myocardial perfusion territory, diffuse atherosclerosis and autoregulation may disproportionately affect resting indices. Finally, studies of microvascular function that integrate CTh-derived flow, resistance and MRR alongside BTh-derived markers are required to further characterize and understand the mechanistic role of microvascular physiology in FFR/RFR discordance and improve cardiovascular outcome among these patients.

## Conclusions

In our study, FFR/RFR discordance was present in over one fifth of physiology studies, was more prevalent in the LAD and was predominantly represented by the FFR−/RFR+ phenotype. CFR was associated with the presence of discordance overall, while within the discordant cohort both CFR and MRR were independently associated with the FFR−/RFR+ phenotype, with low MRR providing numerically greater discrimination than CFR. These hypothesis-generating findings suggest that impaired microvascular vasodilatory reserve, expressed predominantly as resting autoregulatory vasodilation, may be an important mechanistic contributor to the FFR−/RFR+ phenotype, and prospective studies that incorporate the measurement of MRR during epicardial physiology studies are required to better define the role of MRR in guiding phenotype characterization, clinical decision-making and stratified medical therapy.

## Data Availability

The datasets presented in this article are not readily available because they contain confidential patient data. The data, analytic methods and study materials are available from the corresponding author on reasonable request to researchers who meet the criteria for access to confidential data. Requests to access the datasets should be directed to aswin.babu@research.uwa.edu.au.
