# Supplementary material for: Microvascular resistance reserve: insights into discordant resting and hyperemic epicardial coronary physiology
Source: Front Cardiovasc Med. 2026 Oct 14;13:1929694. doi: 10.3389/fcvm.2026.1929694 (PMC13714799; doi:10.3389/fcvm.2026.1929694)
Supplement: Supplementary file 1 [file Datasheet1.docx]

**Supplemental Material**

# **Contents**

**Supplemental Methods**

Pre-specified versus exploratory statistical analysis plan, with brief notes on the per-vessel analytic unit, INOCA exclusion rationale and key protocol specifications (MRR formula, bolus reproducibility threshold).

**Supplemental Tables**

Table S1. Definitions and derivations of common invasive coronary physiology indices.

Table S2. RFR/FFR quadrant distribution in the full vessel cohort (n=891).

Table S3. Discordance prevalence by vessel territory (LAD vs non-LAD).

Table S4. Univariable logistic regression of LAD association with discordance phenotypes.

Table S5. Coronary microvascular dysfunction prevalence and endotypes stratified by concordance status.

Table S6. Transit times and microvascular indices across the four FFR/RFR quadrants (n=210).

Table S7. Univariable logistic regression of candidate predictors of discordance.

Table S8. Exploratory all-indices multivariable logistic regression for any discordance (sensitivity analysis, see Supplemental Methods for caveats given events-per-variable <5 and CFR–MRR collinearity).

Table S9. Exploratory joint logistic regression of basal and hyperemic microvascular resistance for the FFR−/RFR+ phenotype (n=45).

**Supplemental Figures**

Figure S1. Study flow diagram.

Figure S2. Receiver operating characteristic curve for RFR predicting abnormal FFR.

Figure S3. Distribution of coronary microvascular dysfunction endotypes by FFR/RFR quadrant.

# **Supplemental Methods**

**Epicardial physiology acquisition**
Following diagnostic coronary angiography, physiology assessment was performed at the discretion of the treating interventionist to assist clinical decision-making. Intracoronary nitrate (100–200 µg) was administered and the guiding catheter was disengaged briefly from the ostium. A PressureWire X (Abbott Vascular, California, USA) was zeroed and equalized at the tip of a 6 Fr guiding catheter without side-holes and advanced to the distal third of the vessel of interest. Following a period of hemodynamic stability, RFR was recorded as the lowest P_d_/P_a_ averaged over a minimum of four consecutive cardiac cycles at rest. With the pressure sensor held in the same distal position, intravenous adenosine was infused via a peripheral cannula at an initial dose of 140 µg/kg/min, with operator-directed escalation to the maximum pump-rate of 999 µg/kg/min and an infusion duration of at least 90 seconds when required to achieve stable hyperemia. Once stable hyperemic conditions were achieved, FFR was recorded as P_d,hyper_/P_a,hyper_. At the completion of measurements, the pressure sensor was withdrawn to the tip of the guiding catheter and any drift was recorded. Studies with drift ≥ 0.02 were excluded. All tracings were recorded in Coroventis Coroflow (v4 and v5, Coroventis Research AB, Uppsala, Sweden).

**Microvascular physiology acquisition**
Where microvascular assessment was performed, CFR and IMR were measured simultaneously using a bolus thermodilution (BTh) technique (10,11). In brief, three 3 mL boluses of room-temperature saline were injected briskly and consecutively through the guiding catheter at rest to determine the resting mean transit time (T_mn,rest_) and repeated during stable adenosine-induced hyperemia to determine the hyperemic mean transit time (T_mn,hyper_). CFR was calculated as T_mn,rest_/T_mn,hyper_ and IMR as T_mn,hyper_ × P_d,hyper_. Microvascular resistance reserve (MRR) was derived as (CFR × P_a,rest_) / (FFR × P_a,hyper_), in accordance with the definition originally proposed by De Bruyne et al. (9), representing a microcirculatory-specific measurement of vasodilatory capacity. Traces in which the three bolus transit times deviated by more than 20% were repeated where possible, and traces with non-physiological morphology were excluded from the microvascular analysis.

**Pre-specified versus Exploratory Statistical Analyses**

Per-vessel analysis was selected because discordance between FFR and RFR is a vessel-level phenomenon, with physiological indices, microvascular measurements and downstream revascularization decisions all being vessel-specific. Patients investigated for ischemia with non-obstructive coronary arteries (INOCA) without an epicardial stenosis of interest were excluded as the research question required paired epicardial and microvascular indices in vessels in which the interventionist judged hemodynamic assessment to be clinically relevant, and inclusion of INOCA cases would introduce a distinct phenotype in which FFR/RFR agreement would not be clinically meaningful. The primary endpoint (prevalence of FFR/RFR discordance), the diagnostic performance of RFR for detecting abnormal FFR, the clinical and physiological variables associated with discordance phenotypes, and the per-phenotype multivariable logistic regression models adjusted for age, sex and hypertension (main-text Table 3) were pre-specified. Covariate selection was clinically driven rather than data-driven, reflecting established associations with coronary physiology rather than stepwise entry based on univariable thresholds. Missing data were handled by complete-case analysis. Microvascular subset analyses were performed only in vessels with complete CMD data (n = 210). The exploratory all-indices multivariable model reported as Table S8, which enters CFR and MRR simultaneously with the clinical covariate set, was pre-specified as a sensitivity analysis and is not used for primary inference given an events-per-variable ratio below 5 (45 events for 11 predictors) and the mathematical collinearity of the microvascular indices (MRR ∝ CFR/FFR). The per-phenotype multivariable models presented in main-text Table 3, in which each microvascular index is entered separately alongside age, sex and hypertension, constitute the primary inference. Post hoc analysis of transit time (T_mn_), basal microvascular resistance (BMR) and resistive reserve ratio (RRR) and their associations with discordant phenotypes were performed. Receiver operating characteristic (ROC) curves quantifying the discriminative ability of CFR and MRR for each discordant phenotype were constructed using the pROC package (version 1.18.5, Robin et al. BMC Bioinformatics 2011, 12:77) with the area under the curve (AUC) and 95% confidence intervals reported and pairwise comparisons between AUCs performed using the DeLong non-parametric test (DeLong et al., Biometrics 1988). Adjustment for multiple testing was not applied because the reported endpoints were pre-specified and hypothesis-driven rather than discovery-oriented, and a single primary outcome (prevalence of discordance) anchored the analysis. The post hoc analyses described above are likewise unadjusted and should be interpreted as exploratory. No interim analyses or analysis-stopping rules were pre-specified.

# **Supplemental Tables**

**Table S1.**Definitions and derivations of common invasive coronary physiology indices

| **Index** | **Derivation / formula** | **Threshold** | **Physiological interpretation** |
| --- | --- | --- | --- |
| **RFR** | Lowest P_d_/P_a_ averaged over a minimum of four cardiac cycles | ≤ 0.89 | Functionally significant epicardial stenosis demonstrated at rest. |
| **FFR** | P_d,hyper_ / P_a,hyper_ during maximal hyperemia | ≤ 0.80 | Functionally significant epicardial stenosis demonstrated at hyperemia. |
| **CFR** | Mean T_mn,rest_ / Mean T_mn,hyper_ by bolus thermodilution | < 2.5 | Ability of entire coronary circulation to augment blood flow. Combined epicardial and microvascular vasodilatory capacity. |
| **IMR** | T_mn,hyper_ × P_d,hyper_ | ≥ 25 | Hyperemic microvascular resistance, independent of epicardial pressure loss |
| **MRR** | (CFR / FFR) × (P_a,rest_ / P_a,hyper_) | < 3.0* | Microvascular vasodilatory reserve, theoretically independent of epicardial stenosis severity. |
| **CMD** | CFR < 2.5 and/or IMR ≥ 25 | Composite | Composite diagnosis of coronary microvascular dysfunction. |
| **Structural CMD** | CFR < 2.5 and IMR ≥ 25 | Both | Impaired augmentation of blood flow due to elevated microvascular resistance (structural remodeling). |
| **Functional CMD** | CFR < 2.5 and IMR < 25 | CFR only | Reduced resting microvascular resistance with correspondingly high resting flow, limiting further vasodilation. |
| **Isolated IMR elevation** | CFR ≥ 2.5 and IMR ≥ 25 | IMR only | Elevated microvascular resistance despite preserved vasodilatory capacity. |

**Abbreviations:** CFR, coronary flow reserve, CMD, coronary microvascular dysfunction, FFR, fractional flow reserve, IMR, index of microcirculatory resistance, MRR, microvascular resistance reserve, P_a_, aortic pressure, P_a,rest_, resting aortic pressure, P_a,hyper_, hyperemic aortic pressure, P_d_, distal coronary pressure, P_d,hyper_, hyperemic distal coronary pressure, RFR, resting full-cycle ratio, T_mn_, mean transit time, T_mn,rest_, resting mean transit time, T_mn,hyper_, hyperemic mean transit time.

* MRR < 3.0 is the recommended threshold based on pooled data across published MRR cohorts. The index is often reported as a continuous variable and cut-offs may vary.

**Table S2. RFR/FFR quadrant distribution in the full vessel cohort.**

| **Quadrant** | **n** | **%** |
| --- | --- | --- |
| Concordant abnormal (FFR+/RFR+) | 255 | 28.6 |
| Discordant FFR+ (FFR+/RFR−) | 74 | 8.3 |
| Discordant RFR+ (FFR−/RFR+) | 121 | 13.6 |
| Concordant normal (FFR−/RFR−) | 441 | 49.5 |

*Per-vessel distribution across the four FFR/RFR quadrants (n=891). Abnormal FFR defined as ≤0.80, and abnormal RFR defined as ≤0.89.*

**Abbreviations:** *FFR, fractional flow reserve. RFR, resting full-cycle ratio.*

**Table S3. Discordance prevalence by vessel territory.**

| **Territory** | **Vessels** | **Discordant** | **FFR−/RFR+** | **FFR+/RFR−** |
| --- | --- | --- | --- | --- |
| LAD | 528 | 134 (25%) | 92 (17%) | 42 (8%) |
| Non-LAD | 363 | 61 (17%) | 29 (8%) | 32 (9%) |

*Per-vessel rates of any discordance and each phenotype in LAD vs non-LAD territories (n=891).*

**Abbreviations:** *FFR, fractional flow reserve. LAD, left anterior descending. RFR, resting full-cycle ratio.*

**Table S4. Univariable logistic regression: LAD association with discordance phenotypes.**

| **Outcome** | **OR** | **95% CI** | **P value** |
| --- | --- | --- | --- |
| Any discordance | 1.68 | 1.20–2.36 | 0.003 |
| FFR−/RFR+ phenotype | 2.43 | 1.56–3.78 | <0.001 |
| FFR+/RFR− phenotype | 0.89 | 0.55–1.45 | 0.647 |

*Odds ratios from univariable logistic regression with LAD territory as the sole predictor. The FFR−/RFR+ phenotype was significantly over-represented in the LAD territory, whereas the FFR+/RFR− phenotype was not.*

**Abbreviations:** *CI, confidence interval. FFR, fractional flow reserve. LAD, left anterior descending. OR, odds ratio. RFR, resting full-cycle ratio.*

**Table S5. Coronary microvascular dysfunction prevalence and endotypes, stratified by concordance status.**

| **Variable** | **Concordant (n=165)** | **Any discordant (n=45)** | **OR (95% CI)** | **p-value** | **FFR−/RFR+ (n=32)** | **OR (95% CI)** | **p-value** | **FFR+/RFR− (n=13)** | **OR (95% CI)** | **p-value** |
| --- | --- | --- | --- | --- | --- | --- | --- | --- | --- | --- |
| CMD present (any) | 64 (38.8%) | 19 (42.2%) | 1.15 (0.55–2.37) | 0.732 | 15 (46.9%) | 1.39 (0.60–3.19) | 0.434 | 4 (30.8%) | 0.70 (0.15–2.65) | 0.769 |
| Structural CMD | 12 (7.3%) | 7 (15.6%) | 2.34 (0.73–6.97) | 0.137 | 6 (18.8%) | 2.92 (0.82–9.35) | 0.085 | 1 (7.7%) | 1.06 (0.02–8.44) | 1.000 |
| Functional CMD | 36 (21.8%) | 9 (20.0%) | 0.90 (0.35–2.12) | 1.000 | 7 (21.9%) | 1.00 (0.34–2.64) | 1.000 | 2 (15.4%) | 0.65 (0.07–3.20) | 0.738 |
| Isolated IMR elevation | 16 (9.7%) | 3 (6.7%) | 0.67 (0.12–2.49) | 0.770 | 2 (6.2%) | 0.62 (0.07–2.87) | 0.743 | 1 (7.7%) | 0.78 (0.02–5.94) | 1.000 |
| No CMD | 101 (61.2%) | 26 (57.8%) | 0.87 (0.42–1.80) | 0.732 | 17 (53.1%) | 0.72 (0.31–1.67) | 0.434 | 9 (69.2%) | 1.42 (0.38–6.59) | 0.769 |
| MRR < 3.0 | 40 (24.2%) | 14 (31.1%) | 1.41 (0.63–3.05) | 0.344 | 12 (37.5%) | 1.87 (0.76–4.43) | 0.129 | 2 (15.4%) | 0.57 (0.06–2.78) | 0.736 |

*CMD defined as CFR < 2.5 and/or IMR ≥ 25. Endotypes are Structural (CFR < 2.5 with IMR ≥ 25), Functional (CFR < 2.5 with IMR < 25), and Isolated IMR elevation (CFR ≥ 2.5 with IMR ≥ 25). Odds ratios and 95% CIs from Fisher’s exact test (conditional maximum-likelihood estimate). All comparisons are vs the concordant group.*

**Abbreviations:** *CFR, coronary flow reserve. CMD, coronary microvascular dysfunction. FFR, fractional flow reserve. IMR, index of microcirculatory resistance. MRR, microvascular resistance reserve. RFR, resting full-cycle ratio.*

**Table S6. Transit times, microvascular resistance indices and vasodilatory reserve across the four FFR/RFR quadrants (n=210).**

| **Index** | **FFR+/RFR+ (n=37)** | **FFR−/RFR+ (n=32)** | **FFR+/RFR− (n=13)** | **FFR−/RFR− (n=128)** | **p-valueᵃ** | **p-valueᵇ** |
| --- | --- | --- | --- | --- | --- | --- |
| T_mn,rest_, s | 0.73 [0.44–0.96] | 0.60 [0.41–0.80] | 0.80 [0.63–0.97] | 0.76 [0.45–1.10] | 0.248 | 0.101 |
| T_mn,hyper_, s | 0.21 [0.16–0.30] | 0.20 [0.14–0.38] | 0.24 [0.17–0.34] | 0.19 [0.13–0.30] | 0.472 | 0.523 |
| CFR | 2.89 [2.05–4.02] | 2.89 [1.80–4.02] | 3.75 [2.80–3.97] | 3.63 [2.39–5.40] | 0.049 | 0.265 |
| IMR | 12.15 [8.29–15.32] | 13.72 [9.56–21.57] | 16.29 [12.00–21.00] | 14.11 [9.62–21.36] | 0.572 | 0.754 |
| Real BMR, mmHg·s | 52.48 [32.86–73.09] | 42.57 [32.90–70.26] | 70.57 [50.63–94.89] | 64.85 [38.35–95.80] | 0.028 | 0.059 |
| Theoretical BMR, mmHg·s | 60.22 [37.10–83.08] | 46.52 [36.24–75.65] | 73.83 [54.75–95.69] | 66.31 [38.68–100.69] | 0.105 | 0.062 |
| RRR | 4.00 [2.99–5.57] | 3.74 [2.26–4.54] | 4.79 [4.30–7.30] | 4.27 [2.78–6.34] | 0.104 | 0.018 |
| MRR | 4.03 [3.09–5.62] | 3.89 [2.42–4.83] | 4.99 [4.21–7.13] | 4.27 [2.88–6.48] | 0.155 | 0.028 |

Values are median [Q1–Q3]. ᵃKruskal-Wallis test across the four quadrants. ᵇMann-Whitney U test between the two discordant phenotypes (FFR−/RFR+ vs

FFR+/RFR−).

**Abbreviations:** *BMR, basal microvascular resistance. CFR, coronary flow reserve. FFR, fractional flow reserve. IMR, index of microcirculatory resistance. MRR, microvascular resistance reserve. RFR, resting full-cycle ratio. RRR, resistive reserve ratio. Tmn, mean transit time.*

**Table S7. Univariable logistic regression: predictors of FFR/RFR discordance.**

| **Predictor** | **Any discordance** | | | **FFR−/RFR+** | | | **FFR+/RFR−** | | |
| --- | --- | --- | --- | --- | --- | --- | --- | --- | --- |
|  | **OR** | **95% CI** | **p-value** | **OR** | **95% CI** | **p-value** | **OR** | **95% CI** | **p-value** |
| Age (per year) | 1.01 | 0.99–1.03 | 0.187 | 1.04 | 1.02–1.06 | <0.001 | 0.97 | 0.95–0.99 | 0.012 |
| Male sex | 0.90 | 0.63–1.31 | 0.591 | 0.60 | 0.40–0.92 | 0.018 | 2.09 | 1.05–4.14 | 0.035 |
| Hypertension | 1.36 | 0.95–1.93 | 0.090 | 1.68 | 1.08–2.64 | 0.023 | 0.93 | 0.56–1.53 | 0.762 |
| Diabetes mellitus | 1.00 | 0.71–1.39 | 0.979 | 1.28 | 0.87–1.90 | 0.214 | 0.65 | 0.38–1.11 | 0.115 |
| Current/ex-smoker | 1.21 | 0.88–1.66 | 0.249 | 1.10 | 0.75–1.61 | 0.639 | 1.32 | 0.82–2.14 | 0.253 |
| ACS presentation | 0.96 | 0.67–1.37 | 0.818 | 0.88 | 0.57–1.36 | 0.559 | 1.11 | 0.66–1.86 | 0.704 |
| Previous PCI | 1.03 | 0.72–1.49 | 0.855 | 1.03 | 0.66–1.60 | 0.896 | 1.03 | 0.60–1.78 | 0.912 |
| Previous CABG | 4.28 | 1.42–12.89 | 0.010 | 2.89 | 0.88–9.54 | 0.081 | 3.41 | 0.92–12.67 | 0.067 |
| LAD territory | 1.68 | 1.20–2.36 | 0.003 | 2.43 | 1.56–3.78 | <0.001 | 0.89 | 0.55–1.45 | 0.647 |
| CFR (per 1-unit ↑)* | 0.81 | 0.66–0.99 | 0.038 | 0.77 | 0.60–0.98 | 0.034 | 0.94 | 0.69–1.28 | 0.703 |
| MRR (per 1-unit ↑)* | 0.89 | 0.77–1.04 | 0.133 | 0.80 | 0.66–0.97 | 0.025 | 1.09 | 0.89–1.33 | 0.424 |
| IMR (per 1-unit ↑)* | 1.00 | 0.97–1.02 | 0.755 | 1.00 | 0.97–1.02 | 0.826 | 1.00 | 0.96–1.04 | 0.838 |

Per-vessel univariable logistic regression. The any discordance model uses the full vessel cohort (n=891, 195 events). The FFR-/RFR+ and FFR+/RFR- models compare each phenotype with all other vessels, including the opposite discordant phenotype, whereas the models in Table 3 within the main text compare each phenotype with concordant vessels only.

* Microvascular predictors are analyzed in the subset with complete CMD data (n=210 vessels, 45 discordant of which 32 are FFR−/RFR+ and 13 are FFR+/RFR−).

**Abbreviations:** *ACS, acute coronary syndrome. CABG, coronary artery bypass grafting. CFR, coronary flow reserve. CI, confidence interval. FFR, fractional flow reserve. IMR, index of microcirculatory resistance. LAD, left anterior descending. MRR, microvascular resistance reserve. OR, odds ratio. PCI, percutaneous coronary intervention. RFR, resting full-cycle ratio.*

**Table S8. Exploratory all-indices multivariable model for any discordance (sensitivity analysis).**

| **Predictor** | **OR (95% CI)** | **p-value** |
| --- | --- | --- |
| Age (per year) | 1.01 (0.96–1.05) | 0.783 |
| Male sex | 1.61 (0.72–3.82) | 0.258 |
| Hypertension | 1.06 (0.50–2.34) | 0.880 |
| Diabetes mellitus | 1.70 (0.81–3.56) | 0.161 |
| Current/ex-smoker | 0.73 (0.35–1.50) | 0.397 |
| ACS presentation | 0.36 (0.10–1.06) | 0.089 |
| Previous PCI | 0.80 (0.34–1.78) | 0.593 |
| Previous CABG | 1.28 (0.05–15.66) | 0.853 |
| CFR (per 1-unit ↑) | 0.51 (0.28–0.90) | 0.021 |
| MRR (per 1-unit ↑) | 1.41 (0.90–2.21) | 0.127 |
| IMR (per 10-unit ↑) | 0.93 (0.68–1.18) | 0.570 |

Sensitivity analysis in the microvascular subset (n=210 vessels, 45 events) including all clinical covariates and all three microvascular indices simultaneously. IMR scaled per 10-unit increase for interpretability.

**Abbreviations:** *ACS, acute coronary syndrome. CABG, coronary artery bypass grafting. CFR, coronary flow reserve. CI, confidence interval. IMR, index of microcirculatory resistance. MRR, microvascular resistance reserve. OR, odds ratio. PCI, percutaneous coronary intervention.*

**Table S9. Exploratory joint logistic regression of basal and hyperemic microvascular resistance for the FFR−/RFR+ phenotype (n=45).**

| **Predictor** | **OR per SD** | **95% CI** | **p-value** |
| --- | --- | --- | --- |
| Theoretical BMR | 0.41 | 0.17–0.98 | 0.045 |
| IMR | 1.70 | 0.66–4.36 | 0.270 |

Exploratory analysis restricted to the discordant vessels with microvascular data (n=32 FFR−/RFR+ and n=13 FFR+/RFR−), modeling the FFR−/RFR+ phenotype against FFR+/RFR−. Theoretical BMR and IMR are the resting and hyperemic components of MRR (MRR = theoretical BMR/IMR) and were entered simultaneously.

Both terms were standardized and so odds ratios are expressed per standard deviation increase (theoretical BMR 37.0 mmHg·s, IMR 10.8 units).

**Abbreviations:** *BMR, basal microvascular resistance. CI, confidence interval. FFR, fractional flow reserve. IMR, index of microcirculatory resistance. MRR, microvascular resistance reserve. OR, odds ratio. RFR, resting full-cycle ratio. SD, standard deviation.*

# **Supplemental Figures**

**Figure S1. Study flow diagram.**

# Of 1,328 vessels (1,038 patients) screened, 891 vessels (723 patients) met inclusion criteria for the paired FFR/RFR analysis cohort after excluding vessels without paired FFR/RFR data (n=382), vessels from patients enrolled in concurrent trials (n=66), and vessels studied for planned INOCA evaluation (n=57). Exclusion categories overlapped.

**Abbreviations:** *FFR, fractional flow reserve; INOCA, ischemia with non-obstructive coronary arteries; RFR, resting full-cycle ratio.*

**Figure S2. Receiver operating characteristic curve for RFR predicting abnormal FFR (≤0.80).**

AUC 0.861 (95% CI, 0.837–0.886). The Youden optimal threshold (RFR 0.895) and the conventional RFR threshold (0.89) are annotated.

**Abbreviations:** *AUC, area under the curve. CI, confidence interval. FFR, fractional flow reserve. RFR, resting full-cycle ratio.*

**Figure S3. Distribution of coronary microvascular dysfunction endotypes by FFR/RFR quadrant.**

Among the 210 vessels with complete microvascular data, stacked bars show the proportion with no CMD, structural CMD, functional CMD, or isolated IMR elevation within each FFR/RFR quadrant. Structural is defined as CFR < 2.5 with IMR ≥ 25, Functional as CFR < 2.5 with IMR < 25, and Isolated IMR elevation as CFR ≥ 2.5 with IMR ≥ 25. CMD, coronary microvascular dysfunction.

**Abbreviations:** *FFR, fractional flow reserve. IMR, index of microcirculatory resistance. RFR, resting full-cycle ratio.*
